# Cell radiolabeling with acoustophoresis cell washing

**DOI:** 10.1038/s41598-022-13144-x

**Published:** 2022-06-01

**Authors:** Stephen S. Adler, Emmanuel C. Nyong, Raisa A. Glabman, Peter L. Choyke, Noriko Sato

**Affiliations:** 1 Clinical Research Directorate, Frederick National Laboratory for Cancer Research, 9000 Rockville Pike, Building 10, Room B3B51, Bethesda, MD 20892 USA; 2 Molecular Imaging Branch, National Cancer Institute, National Institutes of Health, Bethesda, MD USA

**Keywords:** Microfluidics, Translational research, Molecular imaging

## Abstract

Labeling immune cells with zirconium-89 (^89^Zr)-oxine has become a viable method to track cells in vivo by PET in various pre-clinical animal models and in clinical applications. Currently, ^89^Zr-oxine cell labeling is performed manually, which requires a highly trained specialist and is prone to human error. As the first phase in developing a fully automated radiolabeling system to address this problem, we assess the use of acoustophoresis cell washing to replace the centrifugal cell washing used in the current ^89^Zr-oxine cell radiolabeling procedure. To accomplish this, a cell radiolabeling procedure was developed in which two steps requiring a centrifuge to wash cells were replaced using acoustophoresis cell washing methods. The process was tested using murine EL4 lymphoma and T cells. The centrifuge cell labeling procedure was used as a control to compare the acoustophoresis cell washing procedure. The acoustophoresis method produced radiolabeled cells with similar properties to the centrifugal method when comparing labeling efficiency, labeled specific activity, efficacy of removing unbound ^89^Zr-oxine from the suspension, cell viability measured using annexin V/propidium iodide staining and activation function. This suggests that acoustophoresis cell washing can be used in the design of an automated benchtop, good manufacture practice-qualified acoustophoresis cell radiolabeling device.

## Introduction

Cell-based cancer therapies, wherein cells with anti-cancer properties are extracted from the patient, expanded ex vivo, often genetically engineered to enhance cancer recognition property, such as transduction of T cell receptor^1,2^ or chimeric antigen receptor^3–5^ for adoptive T cell therapy, and then reinjected back into the patient^6,7^ are a new and promising approach. Currently, there are limited tools to monitor this process. One solution is using a novel ^89^Zr-oxine cell labeling method, which enables the patient to be scanned with positron emission tomography (PET)/computed tomography (CT) to ascertain the distribution of the reinfused labeled cells and determine what percentage of the cells are trafficking to the target tissue/organ (e.g. cancer)^8,9^. The standard radiolabeling process requires a series of manual steps which mandate a skilled operator with experience working with cells. ^89^Zr-oxine cell radiolabeling method also relies heavily on centrifugation in preparation and washing the cells throughout the procedure.

The purpose of this research is to test an application of acoustophoresis technology^10^ in a cell radiolabeling procedure. Acoustophoresis uses ultrasound to generate waves in a micro-channel to affect the movement of cells within a fluid, either by trapping and constraining their movement while the fluid flows through the channel^11^ or by moving cells between two different fluids flowing side by side along a micro-channel^12^. This study focuses on using the latter application of acoustophoresis, in which cells are transferred from one solution to another (referred to as acoustophoresis cell washing), replacing the standard cell washing method using a centrifuge (centrifugal cell washing), in a ^89^Zr-oxine radiolabeling procedure^8,9^. Replacing the use of a centrifuge with an acoustophoresis cell washing chip is the first step in exploring the viability of acoustophoresis technology to design a tabletop good manufacturing practice (GMP)-qualified compliant acoustophoresis radiolabeling system. Such a system would simplify the radiolabeling procedure, obsoleting the current manual procedures.

We tested a radiolabeling procedure which uses acoustophoresis cell washing against the current centrifuge-based procedure by measuring the labeling efficiency, specific activity, cell recovery, and labeled cell viability and activation function. We present the comparison results between the two procedures which indicate that the acoustophoresis cell washing procedure has no detrimental effects on the cell radiolabeling process and thus validating the technology to be used in an automated cell radiolabeling system.

## Materials and methods

### Mice

C57BL/6 mice were purchased from Jackson Laboratories and were used to harvest T cells after euthanasia by CO_2_ inhalation. Since mice were not used directly in the study, the ARRIVE principle does not pertain to the work presented here in. Mice were handled in accordance with animal protocols approved by the National Cancer Institute’s Animal Care and Use Committee.

### Cells

EL4 cells (murine T cell lymphoma) were purchased from American Tissue Culture Collection and were grown in RPMI 1640 medium supplemented with 10% fetal calf serum (FCS), 100 IU/ml penicillin, 100 μg/ml streptomycin, and 0.05 mM 2-mercaptoethanol (referred to culture medium hereafter). Subconfluent EL4 cells were centrifuged at 670 xg for 5 min at room temperature and resuspended at 10–20 million cells in 1 ml culture medium for experiments.

Murine T cells were purified from the spleens of mice using magnetic bead separation and expanded ex vivo for 3 days as described in the Supplemental Materials and Methods. T cells resuspended at 10–20 million cells in 1 ml culture medium were used for experiments.

### Cell counting

EL4 cells were counted using a Luna FX7 automated cell counter (Logos Biosystems, South Korea) using Trypan Blue Stain, 0.4% solution (Logos Biosystems, South Korea). T cells were counted using the same stain and an in-house developed cell counting software package written in root (CERN, Switzerland). The software package reads the Luna FX7 images as input and has a manual point and click override feature to allow for manual identification of live and dead cells. This process corrected mistakenly identified cells by the Luna FX7 cell counting software, which had a high misidentification rate for T cells due to their small size.

### Radiolabeling process

The ^89^Zr-oxine cell radiolabeling procedure consists of three main steps: (1) Preparation of the cells in an labeling (incubation) buffer which can be either PBS or a similar non-protein containing solution. (2) Incubation of the cells with ^89^Zr-oxine at a 30:1 cell suspension:^89^Zr-oxine volume ratio. The volume of ^89^Zr-oxine is calculated from the required labeling activity to obtain a target labeled activity in each cell type. (3) Removal of the unbound ^89^Zr-oxine from the incubation buffer using a protein containing solution and then transferring the cells to a solution for suitable use. Plasma-Lyte A (Baxter) with 4% bovine serum albumin (BSA) was used as the final infusion buffer because of its clinical use in infusing immune cells^13^. Steps 1 and 3 involve cell washing.

**Figure 1:**
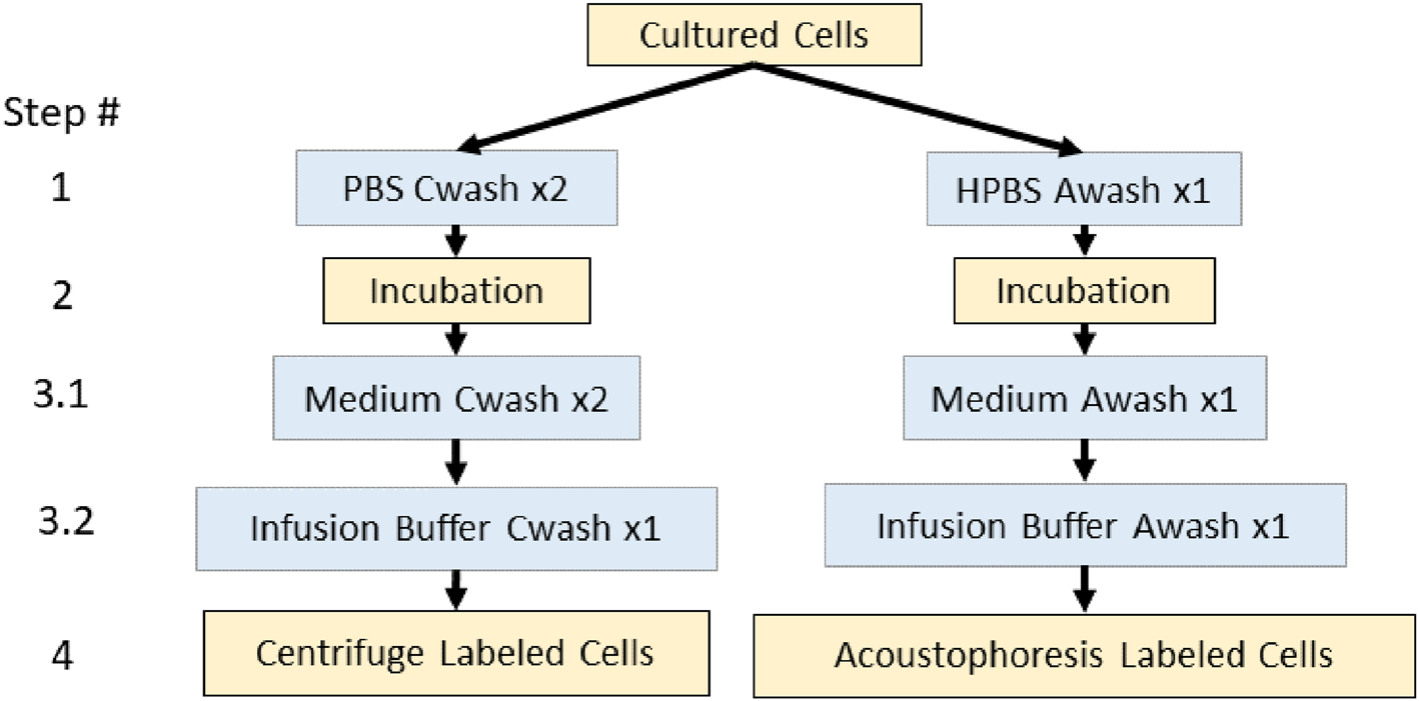
Side by side flow charts of the centrifugation and acoustophoresis cell washing methods. Cultured cells were split into two groups, one labeled using the control centrifugal labeling process while the other labeled using the acoustophoresis process. The centrifuge and acoustophoresis labeling processes were performed concurrently, side-by-side, to provide a best comparison test procedure. The steps in blue are the cell washing steps which could be executed either once or twice as indicated in the flow chart labels (Cwash: centrifugal wash, Awash: acoustophoresis wash).

**Figure 2:**
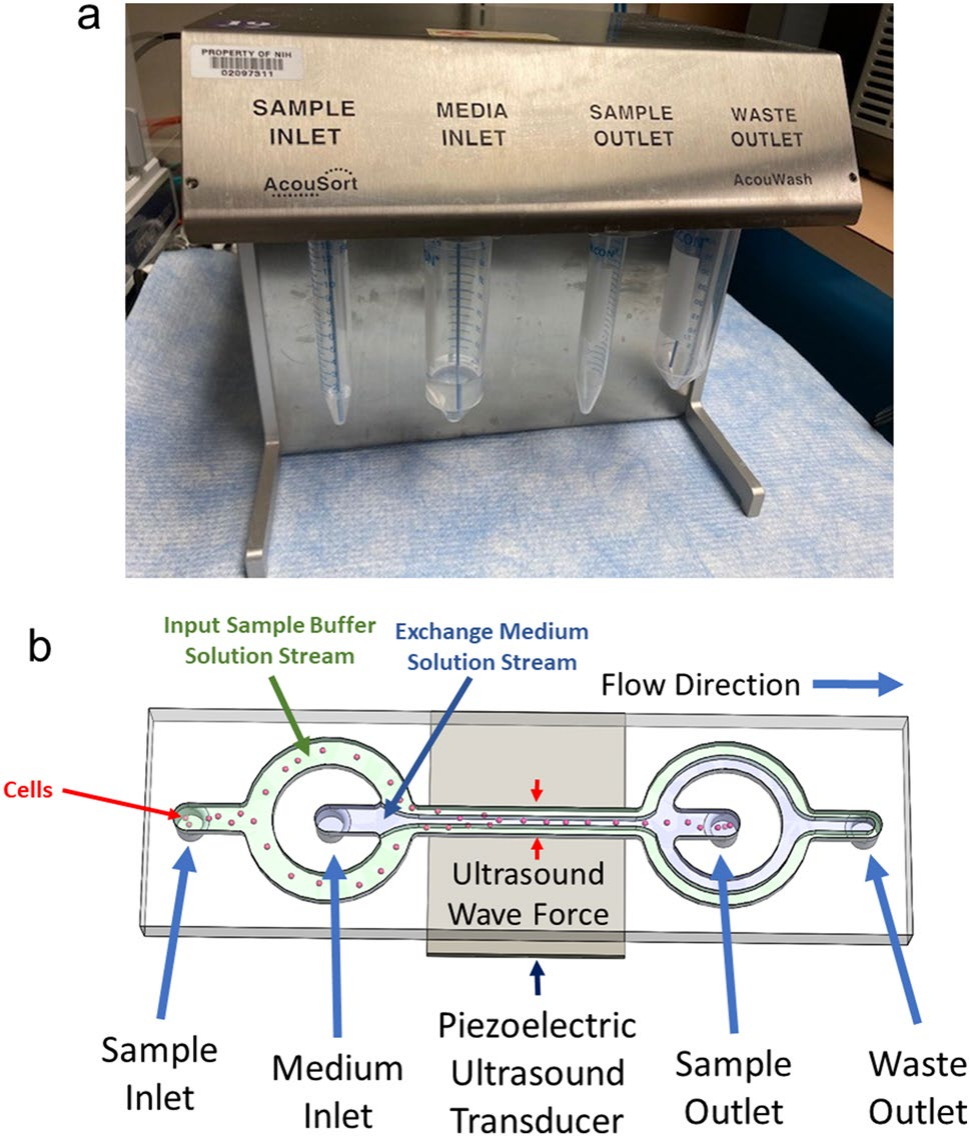
Details of the acoustophoresis cell washing process. (**a**) The AcouWash device used to wash cells using the acoustophoresis cell washing process. The methods section has a full description of its operation. (**b**) A pictorial showing the basic principle of the acoustophoresis cell washing process.

The acoustophoresis and centrifugal washing methods were compared by following the labeling procedures summarized in Fig. 1. An AcouWash system (AcouSort, Lund, Sweden)^14^ was used for acoustophoresis cell washing (Fig. 2a). The device operates by aspirating buffers contained in conical tubes connected to its two inlets. The tube connected to the sample inlet contains the input cells suspended in their original suspension buffer. The tube connected to the media inlet contains the suspension buffer the cells will be transferred to. The fluids from the two inlets are then channeled through the micro-fluidic chip where the cells are transferred from the sample inlet buffer to the media inlet buffer under acoustophoresis forces^12^. See Fig. 2b. For the acoustophoresis cell transfer process to work, the density of the media inlet buffer must be equal to or greater than that of the sample inlet buffer, otherwise the two fluids will mix in the central channel of the micro-fluidic chip ruining the cell washing process^15,16^. The cells transferred into the new buffer solution exit through the sample outlet and a mixture of the two buffer solutions from the two inlets exit through the waste outlet. In the present study, cells suspended in a 1 ml solution was used for the sample inlet and 3–5 ml transfer buffer solution was used for the media inlet.

The wash cycles were performed setting the sample inlet and outlet flow rates to 100 µl/min, the media inlet flow rate to 200 µl/min and setting the wash cycle to high recovery mode. Three wash cycles were performed during the whole acoustophoresis cell labeling process.

In step 1 of acoustophoresis cell radiolabeling procedure, cells suspended in culture medium (1.0124 g/ml) were placed in the sample inlet and a heavy density PBS (HPBS) made by a 20:1 volume ratio mixture of PBS and Lymphocyte Separation Medium (Lonza) (1.0131 g/ml) was placed in the media inlet. The resulting cells suspended in HPBS in the sample outlet were centrifuged at 1500 xg for 3 min and resuspended in HPBS for ^89^Zr-oxine labeling. In the centrifugal cell labeling procedure, cells in the culture medium were spun at 1500 xg for 3 min and the resulting cell pellet was resuspended in PBS for ^89^Zr-oxine labeling. Step 2 of the radiolabeling procedure was performed side-by-side for acoustophoresis and centrifugal procedure samples using the same ^89^Zr-oxine stock solution generated from ^89^ZrCl_4_ as previously described^9^. The estimated radiolabeling efficiency used to calculate the incubation conditions was 30% and 10% for EL4 and T cells, respectively, using the target specific activity of 18.5 kBq/million cells. After the 15 min incubation at room temperature, 1 ml of culture medium was added to the incubation solution, which was typically 100–200 µl in volume.

In the first wash cycle of step 3, a cell suspension in a mixture of HPBS (100–200 µl typical) and 1 ml of culture medium (1.0125 g/ml HPBS + culture medium) was placed in the sample inlet with culture medium (1.0124 g/ml) placed in the media inlet to produce suspended cells in culture medium (step 3.1). In the second wash cycle, cells suspended in culture medium were placed in the sample inlet, with Plasma-Lyte A with 4% BSA (1.0546 g/ml) in the media inlet to produce cell suspension in Plasma-Lyte A with 4% BSA (step 3.2). For step 3 using centrifugal washing, the cells are washed twice with culture medium (step 3.1), then transferred to a new vial upon which the cells are washed a final time with Plasma-Lyte A with 4% BSA (step 3.2).

A final acoustophoresis or centrifuge wash was performed (step 4) in this study from which a sample of the waste or supernatant is aliquoted to measure the remaining % unbound ^89^Zr-oxine post incubation.

### Heavy density PBS determination and laminar flow mixing tests

Step 1 of the labeling process requires cells suspended in culture medium to be washed with PBS. Because the density of PBS (1.0099 g/ml) is lower than that of culture medium (1.0124 g/ml), the laminar flow streams in the acoustophoresis chip mix, ruining the cell washing effect. To solve this problem, PBS solutions with increasing densities were prepared by adding Lymphocyte Separation Medium (Lonza) (1.077 g/ml) at 1:0, 120:1, 60:1, 40:1, 31:1, 25:1 and 20:1 PBS to Lymphocyte Separation Medium volume ratios, corresponding to densities varying from 1.0099 g/ml up to 1.0131 g/ml. Each mixture of heavy density PBS (HPBS, 1 ml) was used in the media inlet and 0.5 ml of culture medium mixed with 37 kBq of ^89^ZrCl_4_ (1–5 µl) in the sample inlet and run through the AcouWash system. The amount of laminar flow mixing was determined by measuring the percent radioactivity in the sample outlet using the equation

1 $$\% \,^{89} {\text{Zr-oxine in outlet}} = \frac{{A_{{{\text{outlet}}}} }}{{A_{{{\text{outlet}}}} + A_{{{\text{waste}}}} }} \times 100\%$$

Similarly, the laminar flow mixing in the first post-incubation washing in the step 3 was examined by placing the post-incubation solution (0.2 ml HPBS, 1.0 ml culture medium, 37 kBq ^89^ZrCl_4_ in 1.2 ml) in the sample inlet and culture medium (3 ml) in the media inlet. For the second post-incubation washing, 0.5 ml culture medium with 37 kBq ^89^ZrCl_4_ was placed in the sample inlet and 1 ml Plasma-Lyte A with 4% BSA in the media inlet. To test whether laminar flow mixing occurs when both the sample inlet and media inlet contain solutions of equal density, PBS was used in both inlets (with ^89^ZrCl_4_ in the media inlet).

### Radioactivity measurements

All radioactivity measurements were performed with a micro-dose calibrator developed in-house, and designed to make accurate radioactive measurements up to 3.7 MBq^17^. All measurements taken were below 0.37 MBq, well within the dynamic range of the micro-dose calibrator.

### Radiolabeling process measurements

Measuring the % unbound ^89^Zr-oxine in the labeled cell suspension was performed differently depending on centrifugal or acoustophoresis labeling procedures. For centrifugation, the ^89^Zr activity and volume of the cell suspension in a 1.5 ml vial were measured, respectively. The cell suspension was then centrifuged at 1500 xg for 3 min. A 0.5 ml aliquot of the supernatant was transferred to an empty 1.5 ml vial and its activity measured. Using the activity concentration of the 0.5 ml supernatant and the original cell suspension volume and activity, the percent unbound ^89^Zr-oxine in the original cell suspension was calculated using the following formula,

2 $$\% \,{\text{unbound 89Zr-oxine}} = \frac{{C_{{{\text{super}}}} V_{{{\text{susp}}}} }}{{A_{{{\text{susp}}}} }} \times 100\%$$

where ${C}_{\text{super}}$ is the activity concentration of the supernatant, ${V}_{\text{susp}}$ is the original cell suspension volume and ${A}_{\text{susp}}$ is the suspension activity.

For the acoustophoresis labeling procedure, because the flow rates averaged over a few seconds are constant between the sample and waste outlets, the activity in the sample and waste outlets were measured. The percent unbound ^89^Zr-oxine was measured using the following formula

3 $$\% {\text{unbound 89Zr-oxine}} = \frac{{A_{{{\text{waste}}}} }}{{A_{{{\text{outlet}}}} + A_{{{\text{waste}}}} }} \times 100\%$$

where ${A}_{\text{outlet}}$ and ${A}_{\text{waste}}$ are the sample and waste outlet activities, respectively.

The labeling efficiency was calculated by taking the incorporated activity and dividing it by the activity measured during the incubation. The cell radiolabeled specific activity (kBq/million cells) was calculated by dividing the incorporated activity by the live cell count. The % cell recovery was calculated by taking the live cell count of the radiolabeled cells and dividing by the starting unlabeled live cell count.

### Flow cytometry analysis

To assess cell viability, cells were stained with 2 µl FITC-conjugated annexin V (BioLegend) and 2 µg/ml propidium iodide (PI, Sigma-Aldrich) in 100 µl of 1X annexin V binding buffer (10X Binding Buffer, R&D Systems) for 15 min in the dark, washed, resuspended in the binding buffer and analyzed on a flow cytometer (CytoFLEX LX, Beckman Coulter). Functionality of ^89^Zr-oxine labeled T cells was assessed via interferon gamma (IFNγ) production after overnight culture with or without stimulation using a plate-coated anti-CD3 antibody (10 µg/ml in PBS, clone 145-2C11) and a soluble CD28 antibody (5 µg/ml, clone 37.51). The T cells were surface stained with anti-CD4-phycoerythrin-cyanine7 (clone RM4-4) and anti-CD8a-fluorescein isothiocyanate (clone 53–6.7) antibodies, followed by intracellular staining using an anti-IFNγ-allophycocyanin antibody (clone XMG1.2) or an isotype control (Rat IgG1-allophycocyanin) and the Intracellular Fixation and Permeabilization Buffer Set (ThermoFisher Scientific) according to the manufacturer’s instructions. The washed cells resuspended in PBS with 0.1% FCS were analyzed via flow cytometery. All antibodies were purchased from ThermoFisher Scientific. The acquired flow data was analyzed using FlowJo software (BD Biosciences).

### Statistical analysis

Two-tailed paired T tests were performed to compare labeled specific activity, labeling efficiency and cell recovery between centrifugal and acoustophoresis radiolabeled cells. A repeated measure one-way analysis of variance (ANOVA) with Tukey’s multiple comparison test, with a single pooled variance, was used to compare the cell viability among the original cells in culture medium and the cells radiolabeled by the centrifugal or acoustophoresis method. A repeated measure two-way ANOVA with Tukey’s multiple comparison test, with a single pooled variance, was used to assess activation of T cells that underwent centrifugal and acoustophoresis radiolabeling methods with and without further activation. GraphPad Prism 8.4.3 (GraphPad Software, San Diego CA) was used for the statistical calculations. *P* values less than 0.05 were considered significant.

All results presented are measured from at least three independently performed experimental procedures. Results of the measurements are presented as means plus standard deviation.

## Results

### Laminar flow mixing

**Figure 3:**
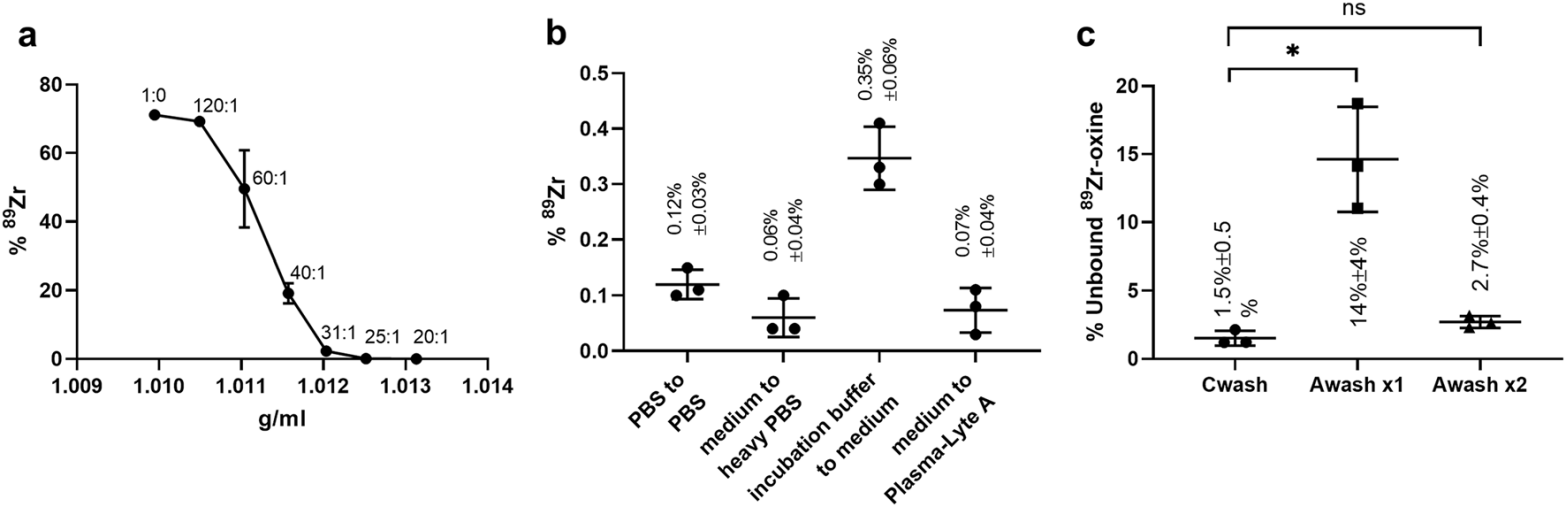
Laminar flow mixing data plots. (**a**) The % ^89^Zr measured in the sample outlet, compared to the activity added in the culture medium in the sample inlet, as a function of various heavy PBS (HPBS) densities used in the media inlet (n = 3, mean ± SD). (**b**) % ^89^Zr measured in the sample outlet for all the acoustophoresis washing steps and also PBS to PBS cell transfer to determine the level of laminar flow mixing using the same density solutions (n = 3, mean ± SD). (**c**) Plots of the % unbound ^89^Zr-oxine remaining in the cell suspension compared to the activity added for incubation, after the complete centrifugal labeling procedure (Cwash) and after one and two wash cycles post-incubation in the acoustophoresis labeling procedure (Awash) (n = 3, mean ± SD, *P < 0.05, ns: not significant).

The results of the laminar flow mixing tests are presented in Fig. 3a,b. When pure PBS was used in the media inlet, the maximal mixing occurred with 71.2 ± 0.6% radiotracer found in the sample outlet. This fell to 0.06 ± 0.04% for the 1:20 HPBS preparation, a low enough radiotracer level to indicate proper operation of the acoustophoresis washing system (n = 3, Fig. 3a). Therefore the 1:20 PBS:Lymphocyte Separation Medium HPBS preparation was used for the remainder of experiments. In all the acoustophoresis washing conditions used throughout the labeling procedure and also in PBS to PBS washing condition, radiotracer levels were less than 0.4% in the sample outlet (n = 3, Fig. 3b).

### Unbound ^89^Zr-oxine removal

Figure 3c shows the % unbound ^89^Zr-oxine remaining in the cell suspension after the complete centrifugal washing procedure and after the first and second wash cycles post-incubation during the acoustophoresis labeling procedure (n = 3). Significantly higher unbound ^89^Zr-oxine was detected after one acoustophoresis wash compared to the centrifugal procedure (*p* = 0.0289), indicating that at least two acoustophoresis wash cycles were required to sufficently remove the unbound ^89^Zr-oxine by acoustophoresis.

### Cell labeling process test results

**Figure 4:**
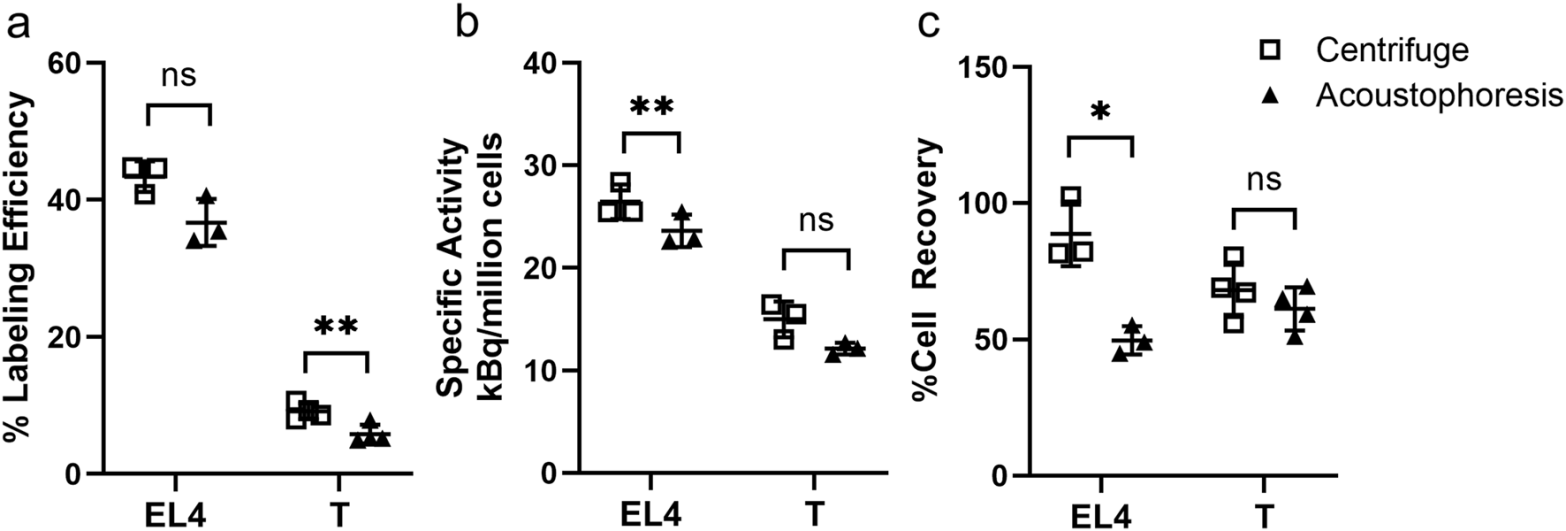
(**a**) Labeling efficiency (the incorporated activity compared to the incubated activity). (**b**) Labeled specific activity (kBq/million cells). (**c**) Cell recovery using centrifugal and acoustophoresis labeling methods. N = 3 for all EL4 cell tests. N = 3 for T cell specific activity and N = 4 for labeling efficiency and cell recovery tests. *P < 0.05, **P < 0.01, ns: not significant. Plots show mean ± SD for each set of N measurements.

The centrifugal and acoustophoresis labeling methods resulted in radiolabeling efficiencies of 43 ± 2% and 37 ± 3% in EL4 cells (*P* = 0.1762, n = 3), and 9 ± 1% and 5 ± 1% in T cells (*P* = 0.0028, n = 3), respectively (Fig. 4a), with specific activity of 26 ± 2 and 23 ± 2 kBq/million cells in EL4 cells (*P* = 0.0010, n = 3), and 15 ± 2 and 12 ± 1 kBq/million cells in T cells (*P* = 0.0722, n = 4), respectively (Fig. 4b). Percent cell recovery was 88 ± 12% and 50 ± 5% for EL4 cells (*P* = 0.0115, n = 3) and 68 ± 10% and 61 ± 8% for T cells (*P* = 0.4003, n = 4), for centrifugal and acoustophoresis labeling methods, respectively (Fig. 4c). Of note, purities of T cells used in the experiments were greater than 90% determine by flow cytometry analysis.

### Cell viability and functionality post labeling

**Figure 5:**
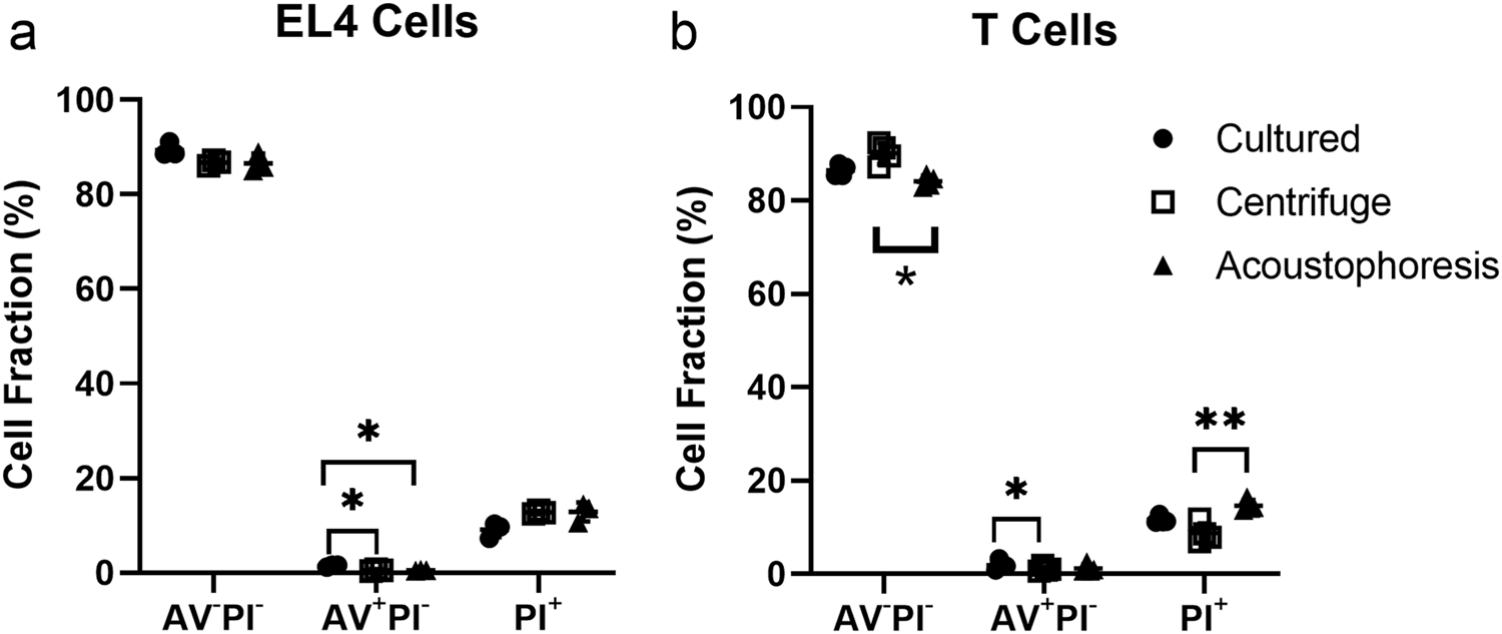
Annexin V (AV) and PI staining of the EL4 cells (**a**) and T cells (**b**) in the original cell culture (cultured) and after full ^89^Zr-oxine labeling procedure using centrifugal (centrifuge) and acoustophoresis methods, analyzed with flow cytometry (n = 3). The AV^−^PI^−^ cells are considered viable, while the AV^+^PIs^−^ cells are early apoptotic, AV^−^PI^+^ and AV^+^PI^+^ cells (PI^+^) are necrotic or late apoptotic. Only statistically significant differences between samples are annotated in the plots (n = 3, *P < 0.05, **P < 0.01). Plots show mean ± SD for each set of N measurements.

Cells which did not stain with annexin V nor PI are considered viable. EL4 cells in culture, and after the centrifugal and acoustophoresis labeling, had a viability of 89 ± 12%, 87 ± 1% and 87 ± 2%, respectively (overall *P* = 0.1721, n = 3, Fig. 5a). For T cells, the viability was 86 ± 1%, 90 ± 2% and 84 ± 1% (overall *P* = 0.0123, n = 3, Fig. 5b) for the cultured, and centrifuge and acoustophoresis labeled cells, respectively. The viability of the cultured and acoustophoresis labeled T cells did not show significant difference (*P* = 0.2758).

**Figure 6:**
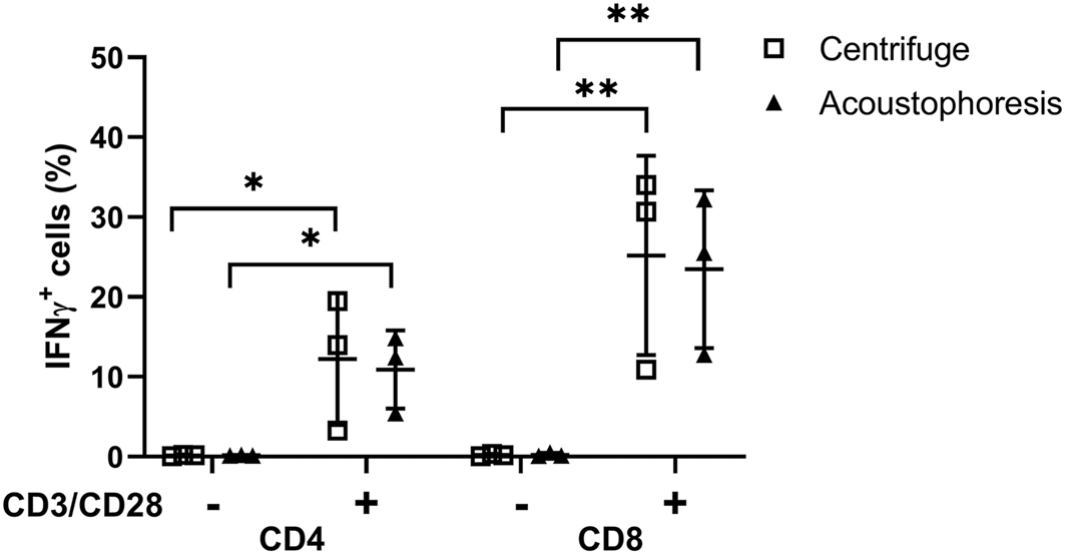
IFNγ production in T cells following centrifugal and acoustophoresis labeling methods. ^89^Zr-oxine labeled T cells without (−) and with (+) overnight CD3/CD28 stimulation were examined for their IFNγ production within CD4 and CD8 T cell populations (n = 3, *P < 0.05, **P < 0.01). The data show that the centrifugal and acoustophoresis labeling processes demonstrate similar cytotoxic function. Plots show mean ± SD for each set of N measurements.

Figure 6 summarizes IFNγ production function of T cells labeled with centrifugal and acoustophoresis methods upon an activation through the T cell receptor (n = 3). Without additional CD3/CD28 stimulation, CD4 and CD8 T cells did not express IFNγ: 0.16 ± 0.10% and 0.17 ± 0.06% of CD4 T cells and 0.21 ± 0.15% and 0.24 ± 0.22% of CD8 T cells labeled with the centrifuge and acoustophoresis procedures, respectively, expressed IFNγ (Fig. 6, Fig. S2). A CD3/CD28 stimulation significantly induced IFNγ expression in both CD4 and CD8 T cells: 12 ± 8% (*P* = 0.0319) and 11 ± 5% (*P* = 0.0400) of CD4 T cells and 25 ± 12% (*P* = 0.0192) and 23 ± 10% (*P* = 0.0221) of CD8 T cells labeled with the centrifuge and acoustophoresis procedures, respectively, produced IFNγ (*P* values represent comparison between non-stimulated vs stimulated cells). Importantly, acoustophoresis labeled T cells possessed similar IFNγ production function upon activation as the cells underwent the centrifugal labeling procedure.

## Discussion

The goal of this study is to determine if acoustophoresis cell washing could substitute for centrifugal cell washing in the ^89^Zr-oxine cell radiolabeling procedure. Cell washing is a critical component of the radiolabeling procedure (e.g. preparing the cells for labeling and removing the unbound tracer) and validating the use of acoustophoresis washing is an important step in determining the feasibility of designing a fully automated acoustophoresis cell radiolabeling system for GMP applications.

The validation tests were designed to compare several measurements between the acoustophoresis cell washing process and the centrifugal washing procedure which characterize how well the labeling procedure worked. These are the labeling efficiency, labeled cell specific activity, cell recovery, cell viability, cell activation and the percent unbound ^89^Zr-oxine remaining in the labeled cell product suspension. To best compare the acoustophoresis labeling process to the control centrifugal process, two cell samples were harvested from the same culture flask, with one sample labeled using the acoustophoresis process and the other labeled using the centrifugal process. The two samples underwent the labeling procedure side-by-side throughout the labeling process, including the use of the same stock ^89^Zr-oxine solution on both samples at the incubation step.

When developing the acoustophoresis radiolabeling procedure, the data indicated that the buffers used to wash the cells post-incubation had a significant effect on cell viability. After initial testing, documented in the supplemental material, it was discovered that to maximize cell viability, the first post-incubation acoustophoresis cell wash was optimized with culture medium. This should be followed by washing the cells in the final suspension buffer suitable for cell usage.

The ability to remove the free unbound ^89^Zr-oxine from the incubation suspension by the acoustophoresis washing method using 2 wash cycles was similar to the centrifuge washing process. The cells labeled using the acoustophoresis procedure were similar to the centrifuge procedure in their specific activity, cell viability and cellular function. While there may have been deviations between the two procedures, some significant under statistical analysis, the size of the differences are small enough that none of the differences invalidate the use of acoustophoresis washing in designing and implementing an automated acoustophoresis radiolabeling system. Together, these measurements demonstrate that acoustophoresis cell washing in the radiolabeling procedure is not detrimental to the cell labeling process or to the cells and T cell activation properties. We consider the principle of acoustophoresis cell radiolabeling procedure presented using ^89^Zr-oxine in this study to be applicable to other cell labeling agents, such as ^89^Zr conjugated antibody cell labeling for PET^18^ and ^111^In-oxine cell labeling for single photon emission tomography imaging^19^.

One limitation of this work is that it was only able to test the cell washing steps of the labeling process and did not achieve a full acoustophoresis-only cell labeling process. The cells must be concentrated for incubation with ^89^Zr-oxine and centrifugation was used for this step in the acoustophoresis branch of the labeling process. Future work will focus on developing acoustophoresis methods to concentrate the cells appropriate for radiolabeling, which allows an acoustophoresis-only cell labeling procedure without relying on centrifugation.

Another limitation of this work was that the cell recovery was inconsistent and worse for the acoustophoresis labeling process compared to the centrifugation method. This is due to a relatively long tubing used in the AcouWash system creating a large “swept volume” of approximately 200 µl as compared to the 1 ml cell sample volume which was used in the washing process. A properly designed multi-step acoustophoresis labeling system would improve the cell recovery.

Future studies will focus on the up-concentration of cells to the cell concentration needed for incubation to achieve a fully acoustophoresis cell radiolabeling process. Further work will focus on optimizing cell washing flow rates and cell concentration levels with a goal to minimize the number of acoustophoresis cell washing cycles and maximizing cell radiolabeling throughput.

## Conclusion

The use of acoustophoresis cell washing, replacing the centrifugal washing, in the ^89^Zr-oxine cell radiolabeling procedure has been validated. The results indicate that acoustophoresis cell washing is a viable technology and can be used for the washing phase when designing a countertop all-in-one cell radiolabeling device. The next phase of this research project will be to integrate acoustophoresis methods to prepare cells at the correct cell concentration needed for incubation with a radiotracer and re-evaluate the full procedure. If successful, an acoustophoresis bench top system to radiolabel cells can be designed. The benefit of such a device is that it removes many of the critical manual pipetting steps thus removing human error and increasing the reliability of the cell radiolabeling process. Furthermore, it would integrate with ease into a GMP radio-pharmacy.

## Supplementary Information

Supplementary Information.

## Data Availability

The datasets generated during and/or analyzed during the current study are available from the corresponding author on reasonable request.
