# Supplementary material for: Cell radiolabeling with acoustophoresis cell washing
Source: Sci Rep. 2022 Jun 1;12:9125. doi: 10.1038/s41598-022-13144-x (PMC9160075; doi:10.1038/s41598-022-13144-x)
Supplement: Supplementary file 1 — Supplementary Information. [file 41598_2022_13144_MOESM1_ESM.docx]

Cell Radiolabeling with Acoustophoresis Cell Washing

Supplemental material

# Murine T cell purification

The spleens harvested from mice were passed through a 70 µm-pore strainer (Falcon). The obtained single cell suspension was layered over Lymphocyte Separation Medium (Lonza) and centrifuged at 510 x g for 12 minutes with low acceleration/deceleration at room temperature. The cells in the interphase were collected, washed with phosphate buffered saline (PBS), and incubated with CD8a MicroBeads and then with CD4 MicroBeads (Miltenyi), following the manufacturer’s instruction. The cells were then washed with PBS and a mixture of CD8 and CD4 T cells were purified using an autoMACS Pro separator (Miltenyi). The purified T cells were weakly activated by adding 250 µl of Dynabeads Mouse T-Activator CD3/CD28 (ThermoFisher Scientific) and 0.2 nM human IL-2 (Peprotech) to 5x10^7^ cells in 15 ml culture medium. One day and two days later, 10 ml and 25 ml culture medium was added, respectively. Three days later, the cells were collected, washed with PBS, and resuspended in culture medium at 10-20 million cells in 1 ml for experiments.

# Cell viability versus washing solutions study

The initial full radiolabeling tests performed using acoustophoresis cell washing resulted in poor relative cell viability, which suffered from a decrease in cell viability of up to 14% in EL4 cells (Supplemental Figure 1a) when comparing the cell viability of the labeled cells (data labeled “AcouWash” for acoustophoresis labeled cells, Supplemental Figure 1a) to the viability of the starting cell sample (data labeled “Cultured”, Supplemental Figure 1a.) The cells labeled using the centrifuge method showed no measurable decrease in viability (data labeled “Centrifuge”, Supplemental Figure 1a.) The initial procedure was to wash cells only with HPBS at all stages of the labeling procedure, both in the initial cell wash which prepares cells for incubation and in the post-incubation washes which removes the excess ^89^Zr-oxine through two acoustophoresis cell cycles. A breakthrough in viability occurred when instead of performing two post-incubation acoustophoresis wash cycles in HPBS, the cells were washed twice in culture medium. This resulted in a statistically insignificant drop in viability of -2.2%±0.6% (P=0.1366 by overall repeated-measure one-way ANOVA, Supplemental Figure 1b) relative to the viability of the initial culture cell sample. From this it was determined that the final cell suspension solution should have a protein rich environment, and therefore Plasma-Lyte A with 4% BSA was used as the final suspension solution for the radiolabeled cells. This solution was chosen because it is used to infuse immune cells in clinical settings. Further testing in which HPBS was used in the first post-incubation acoustophoresis wash and Plasma-Lyte A with 4% BSA for the second acoustophoresis wash still exhibited decreased cell viability (Supplemental Figure 1c). The final set of tests were performed using culture medium for the first acoustophoresis cell wash post-incubation, and Plasma-Lyte A with 4% BSA for the second acoustophoresis cell wash. This resulted in good cell viability for which a statistically insignificant drop of -2%±12% (P=0.1721 by overall repeated-measure one-way ANOVA, Supplemental Figure 1d) was observed. The use of culture medium for the first acoustophoresis wash followed by Plasma-Lyte A with 4% BSA for the second acoustophoresis wash was thus adopted into the acoustophoresis cell radiolabeling procedure.

To note, the data presented in Supplemental Figure 1 shows the cell viability of the initial cell sample which is labeled “Culture”, the cell viability of the cells which underwent the centrifuge based radiolabeling procedure labeled “Centrifuge” and the cells which underwent the acoustophoresis based radiolabeling procedure labeled “AcouWash”. This set of data is presented for the four different combinations of buffer solution showing the method of optimizing cell viability for the acoustophoresis radiolabeling process.

| 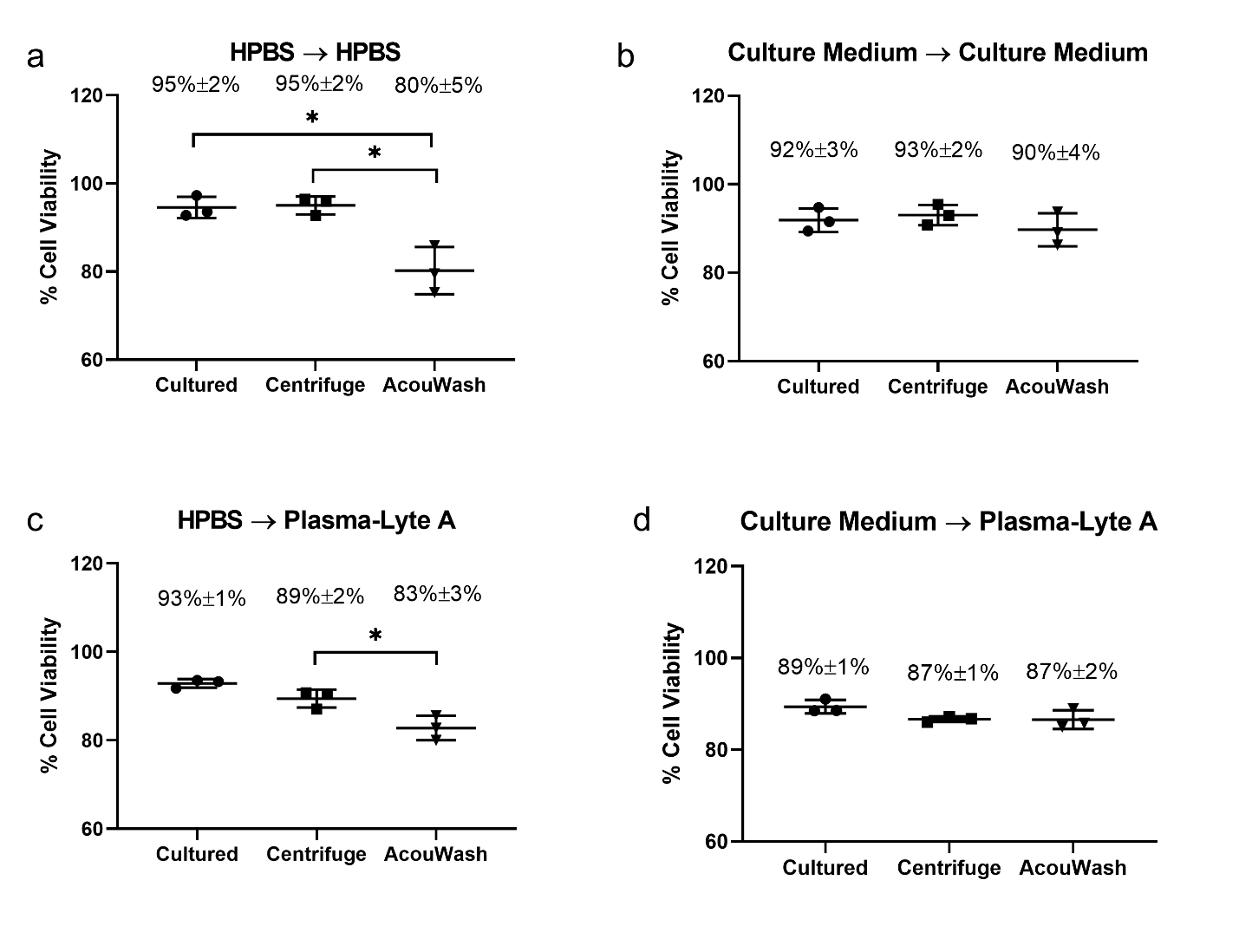 |
| --- |
| SUPPLEMENTAL FIGURE 1. Cell viability measured using annexin V/PI staining analyzed by flow cytometry. The cells that did not stain with annexin V nor PI were considered viable. a-d show the various attempts at optimizing the sequence of post-incubation acoustophoresis cell washing solutions. a) plots the viability data when HPBS solution was used in both post-incubation acoustophoresis cell washing steps. b) plots the viability data when culture medium was used in both post-incubation acoustophoresis cell washing steps. c) plots the viability when HPBS was used for the first acoustophoresis cell wash post-incubation followed by a Plasma-Lyte A with 4% BSA cell wash. d) plots the cell viability when the cells post-incubation were acoustophoresis washed with culture medium followed by Plasma-Lyte A with 4% BSA. Only differences which were statistically significant are marked (n=3, *:p<0.05 repeated-measure one-way ANOVA). The viability labeled Cultured is the viability of the starting cell sample before commencing radiolabeling. The viability labeled Centrifuge is the viability measurements of the cells underwent the centrifuge based radiolabeling procedure. The viability labeled “AcouWash” is the viability measurements of the cells underwent the acoustophoresis based radiolabeling procedure. Plots show mean ± SD for each set of N measurements. |

| 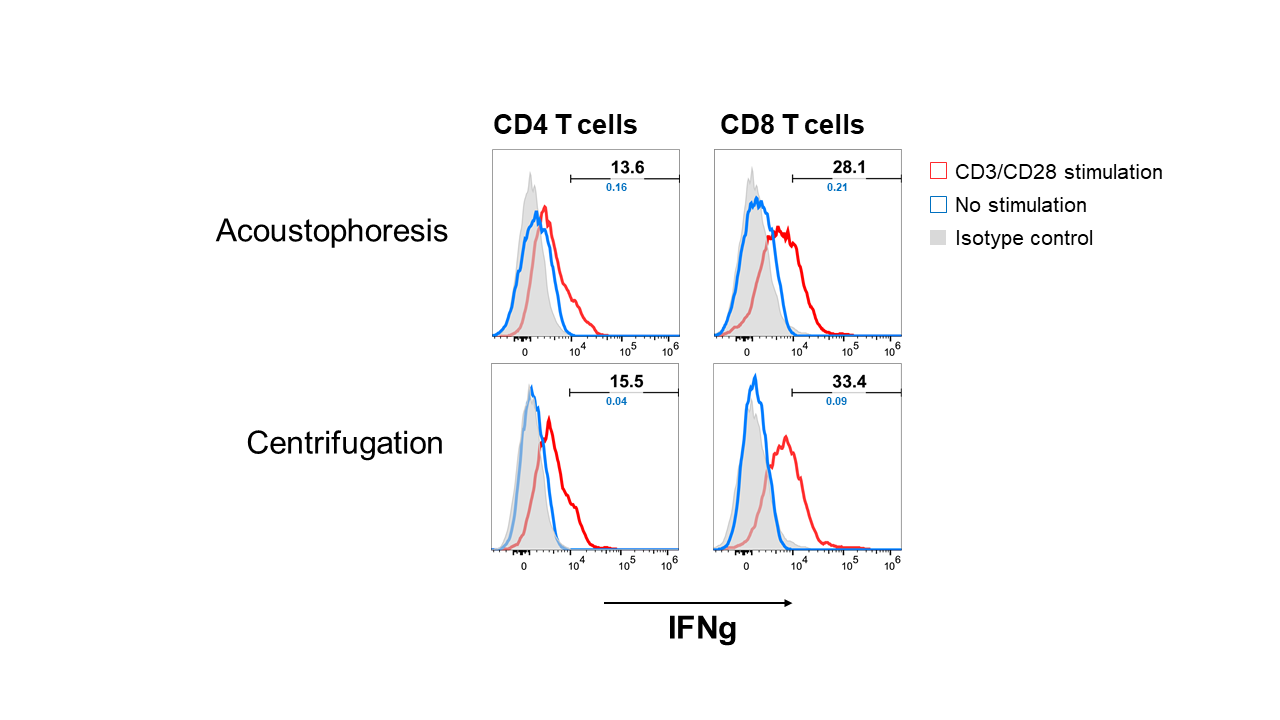 |
| --- |
| SUPPLEMENTAL FIGURE 2. Representative flow cytometry data of IFNγ expression in CD4 and CD8 T cells labeled with ^89^Zr-oxine via acoustophoresis labeling or centrifugal labeling procedures, with or without CD3/CD28 stimulation for overnight (n=3). The percentage of IFNγ positive cells for CD3/CD28 simulated cells are shown above the marker and those for non-stimulated cells are shown below the marker in each plot. See Figure 6 for the statistical analysis results. |
